# Exploring the Potential of Syngas Fermentation for Recovery of High-Value Resources: A Comprehensive Review

**DOI:** 10.1007/s40726-024-00337-3

**Published:** 2024-11-21

**Authors:** Alvaro S. Neto, Steven Wainaina, Konstantinos Chandolias, Pawel Piatek, Mohammad J. Taherzadeh

**Affiliations:** 1 Swedish Centre for Resource Recovery, University of Borås, Borås, Sweden; 2 Division of Built Environment, RISE Research Institutes of Sweden, Gothenburg, Sweden; 3 Millow AB, Gothenburg, Sweden

**Keywords:** Syngas fermentation, Mixed bacteria, Biofuels, Industrial scale, Conditioning methods, Process development

## Abstract

Synthesis gas (syngas) fermentation represents a promising biological method for converting industrial waste gases, particularly carbon monoxide (CO) and carbon dioxide (CO₂) from industrial sources (e.g. steel production or municipal waste gasification), into high-value products such as biofuels, chemicals, and animal feed using acetogenic bacteria. This review identifies and addresses key challenges that hinder the large-scale adoption of this technology, including limitations in gas mass transfer, an incomplete understanding of microbial metabolic pathways, and suboptimal bioprocess conditions. Our findings emphasize the critical role of microbial strain selection and bioprocess optimization to enhance productivity and scalability, with a focus on utilizing diverse microbial consortia and efficient reactor systems. By examining recent advancements in microbial conditioning, operational parameters, and reactor design, this study provides actionable insights to improve syngas fermentation efficiency, suggesting pathways towards overcoming current technical barriers for its broader industrial application beyond the production of bulk chemicals.

## Introduction

In recent years, efforts to mitigate and address global warming have increasingly emphasized the urgent need to enhance resource efficiency, while significantly reducing societal dependence on fossil fuels. For instance, the share of renewable energy consumed in the European Union has achieved 23% driven by the establishment of new energy sources, such as solar power, and the reduced use of non-renewable sources [1]. Moreover, during COP28, in the global renewables and efficiency pledge, 133 countries have declared to work and pursue three times more renewable energy capacity by 2030 [2].

There are several examples of renewable energy resources available for transition to a circular economy, such as solar, wind, and biomass. Looking towards circular economy development, biomass is an important resource for sustainable, carbon-negative chemical and energy production from waste materials which can be derived from plants, animals, microorganisms, and wastes or residuals, i.e. agricultural, forestry, industrial, and municipal [3].

Different types of processes have been proposed for the conversion of biomass, such as thermochemical, biological, and a hybrid thermochemical/biochemical process. Some examples of thermochemical processes are gasification and pyrolysis. During gasification, the biomass is converted into synthesis gas (or syngas) which is mainly composed of carbon monoxide (CO), hydrogen (H_2_), and carbon dioxide (CO_2_), along with residual gases such as methane (CH_4_), ethylene, ethane, hydrogen sulphide (H_2_S), and ammonia, and other impurities as tar, ash, and solid char [4]. Pyrolysis is a thermal treatment process where biomass is converted in an inert atmosphere at medium to high temperature into products with higher energy density, such as bio-oil, biofuel, syngas, and bio-char. [4].

Anaerobic digestion is one of the possible conversion routes of biomass where different waste streams can be used as feedstock (food waste, organic municipal solid waste, sewage sludge, etc.) and be converted in the absence of oxygen to CO_2_ and CH_4_ [3]. Given the abundant source of the feedstock, anaerobic digestion is an integrated, cost-effective process in producing value-added renewables products, such as biogas, H_2_, alcohols, and volatile fatty acids (VFAs), which have significant utility within a circular bioeconomy [5]. Combining thermochemical and biological conversion of biomass requires the utilization of gas fermentation, where thermochemical gasification creates a gas mixture that serves as a feedstock for the fermentation process, that employs pure cultures, co-cultures, or a natural consortium from diverse sources of anaerobic sludges (sewage, wastewater treatment, food waste, municipal solid waste, lignocellulosic biomass, etc.), allowing product of diverse chemical products such as biofuels and chemicals that could supplement or even replace current fossil-based commodities [6].

Widespread use of syngas fermentation on an industrial scale still faces challenges through several factors, such as high up-front scale-up costs, low gas-to-water mass transfer which limits carbon (CO/CO_2_) and electron (H_2_) availability for microbial biocatalysts, and relatively slow microbial growth rates and product outputs [7]. Nevertheless, these disadvantages could be overcome with the advancement of genetic and metabolic engineering, coupled with novel bioprocess developments. With an increasingly clear understanding of the metabolism of these microorganisms applied to critical parameter changes, such as temperature, pH, microbial consortium characterization, gas mixing, and gas composition, improvements can be achieved, which will become the focus of this review. Gas fermentation has been a research focus for several decades and has accelerated in the last 15 years due to becoming a viable and sustainable industrial application for next-generation bio-commodities. Reviews have scrutinized its drawbacks, such as reactor designs [8], critical operational parameters [9, 10], and importantly the integral microbial mechanisms of gas metabolism [11]. Previous reviews conducted over the past few years have predominantly focused on alcohol production [10, 12], which is an industry-scale process now. Other aspects, such as the production of diverse products (e.g. H_2_ and organic acids) and the application of mixed cultures and their conditioning methods on an industrial scale, have been overlooked. To facilitate an interdisciplinary discussion, this paper systematically reviews the interplay between biological processes and relevant engineering advancements. It provides valuable insights and examples to enhance the application of syngas fermentation on larger scales for sustainable biofuel and chemical production. The review covers relevant publications, primarily from January 2019 to November 2024. Several approaches to address the limitations of syngas fermentation are discussed and exemplified to overcome current research barriers and facilitate broader industrial application of this process.

## Overview of the Syngas Fermentation and Potential Products

**Fig. 1:**
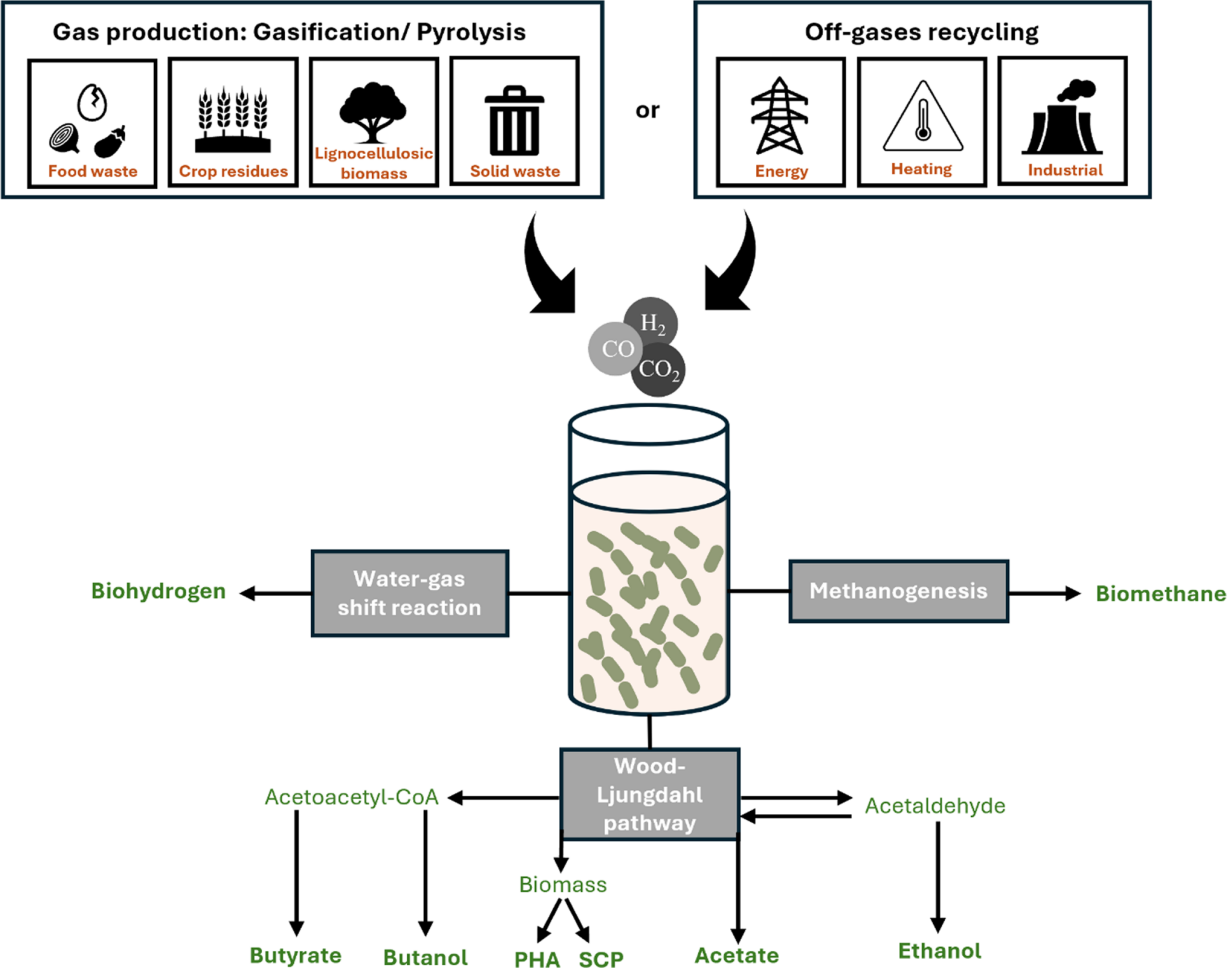
Overview of syngas fermentation process from diverse gas feedstocks and possible end-products by water–gas shift reaction process, methanogenesis, and Wood-Ljungdahl pathway metabolism. PHA: polyhydroxyalkanoates. SCP: single-cell protein

Syngas fermentation is a biological process where waste gas streams from thermochemical or industrial processes are converted into value-added products (Fig. 1). From thermochemical processes, a variable mixture of H_2_, CO, and CO_2_ (syngas) is produced with fractions dependent on the type of biomass used as a thermochemical feedstock, operational parameters, and oxidizing agents [10]. Moreover, waste gases generated from specific heavy industries, such as steel mills, can have more predictable CO-rich streams and have been the focus for initial investment for gas fermentation scale-up by companies (e.g. Lanzatech) [13]. Raw syngas also contain impurities (e.g. sulfuric acid, ammonia, tar, ash) related to biomass properties and gasification conditions, which have a direct impact on bacterial activity, possessing potentially toxic effects on some species [14]. However, one of the advantages of syngas fermentation is the microbial adaptability in contrast with syngas conversion via chemo catalysis, where microorganisms are less prone to be poisoned by gas impurities than chemical catalysts [15]. Moreover, microorganisms can be gradually adapted to inhibitory syngas impurities, presenting similar productions as those without facing inhibition by the impurities compounds [16].

Syngas fermentation can occur by four main groups of microorganisms: methanogenic archaea, hydrogenogenic bacteria, sulphate-reducing bacteria, and acetogenic bacteria [17]. These microorganisms can be classified as carboxydotrophic microorganisms capable of oxidizing CO for energy conservation and growth [18]. Therefore, these microorganisms are ubiquitous in many environments, presenting diverse optimum growth conditions and product formation [11]. Focus on specific strains of gas fermenting species has generated a wealth of knowledge ranging from thermodynamic and metabolic understanding and a means of genetically editing towards desired fermentation products. This contrasts with mixed cultures, where difficulty in performing the same in-depth studies remains a challenge. However, the use of a natural consortium is still a viable and under-explored option to scale cost-effectively due to the significantly lower sterility requirements [18]. Ethanol is one of the most targeted products derived from syngas fermentation and is currently produced on an industrial scale [7]. However, many other products can be produced from the syngas fermentation process, such as chemicals (e.g. acetic acid, propionic acid, butyric acid), alcohols (e.g. butanol, isopropanol), and gases (e.g. CH_4_, H_2_) [11, 19–21].

### Microbial Pathways

In the syngas mixture, CO is one of the main gases present, and despite being highly toxic to most living creatures, some anaerobic microorganisms can survive and convert CO into different products [22]. Anaerobic microorganisms utilize CO via the enzyme carbon monoxide dehydrogenase (CODHs) in their fermentative process, with different metabolisms, such as acetogenesis (Eq. 1), methanogenesis (Eq. 2), hydrogenogenesis/biological water–gas shift reaction (Eq. 3), and solventogenesis (Eq. 4). [23]. The conversion of CO in anaerobic microorganisms is catalyzed by nickel–iron (Ni, Fe)-CODHs, an oxygen-sensitive enzyme [24]. CODH is responsible for catalyzing the reversible oxidation of CO to CO_2_ via Eq. 3.

1 $$4\text{ CO}+{2\text{ H}}_{2}\text{O}\to 2{\text{ CO}}_{2}{\text{COO}}^{-}+ {\text{H}}^{+}+2{\text{ CO}}_{2}$$

2 $$4\text{ CO}+{2\text{ H}}_{2}\text{O}\to {\text{CH}}_{4}+3{\text{ CO}}_{2}$$

3 $$\text{CO}+{\text{H}}_{2}\text{O}\to {\text{CO}}_{2}+{\text{ H}}_{2}$$

4 $$6\text{ CO}+{3\text{ H}}_{2}\text{O}\to {\text{C}}_{2}{\text{H}}_{5}\text{OH}+4{\text{ CO}}_{2}$$

During CO oxidation, electrons are generated by CODH and used in various reductive processes. In the case of anaerobic reactions, these electrons can be used to generate acetate from CO_2_ (acetogenesis) and then CH_4_ from acetate (methanogenesis). In addition, a proton reduction can generate H_2_ (hydrogenogenesis/biological water–gas shift reaction) from this process. Moreover, acetogenic bacteria can reduce acetyl-CoA into acetaldehyde and then to ethanol via the Wood-Ljungdahl pathway (solventogenic) or via acetate synthesis [23, 25].

#### Biological Water–Gas Shift Pathway

The most common means to produce H_2_ is from steam reforming processes, where a mixture of steam and hydrocarbons reacts to produce H_2_, CO, and CO_2_. Through the water–gas shift (WGS) reaction, CO reacting with water is converted to CO_2_ and additional H_2_ [26]. The WGS reaction is a reversible exothermic reaction as described in Eq. 5:

5 $$\text{CO}+{\text{H}}_{2}\text{O }\leftrightarrow \text{ C}{\text{O}}_{2}+{\text{H}}_{2}$$

WGS reactions are also performed biologically, and instead at elevated temperatures of steam reforming processes, WGS can also occur at mesophilic temperatures. Previous studies have reported nearly a total conversion of CO into H_2_ in the syngas fermentation process [21, 27]. Different microorganisms have been reported to produce H_2_, such as aerobic bacteria (e.g. *Bacillus*), restricted anaerobes (e.g. *Clostridium*, *Thermoanaerobacterium*, *Caloramator*), and archaea (e.g. *Thermococcus onnurineus*) [28, 29]. For these microorganisms, CO oxidation and proton reduction are simple biological mechanisms to conserve energy [23]. During the biological WGS reaction, three different enzymes are responsible for H_2_ production and carbon fixation by hydrogenic organisms [30]. First, CO is oxidized by CODH releasing electrons that are transferred to a “ferredoxin-like” electron carrier. CODH retains a Ni–Fe active site that has a strong tendency to be bound to CO, inhibiting the hydrogenase activity. In this way, a high tolerance to CO by the hydrogenase coupled with CODH needs to be present to avoid the inactivation of the catalytic cycle. Furthermore, the energy-conserving-hydrogenase (EcH) enzyme oxidizes the electron carrier coupled with a two-proton reduction to produce H_2_ [22, 23, 30, 31]. Overall, the biological WGS reaction to produce H_2_ utilizing CO can be described as in Eq. (3) previously noted.

#### Wood-Ljungdahl Pathway

Acetogens are anaerobic bacteria able to use the Wood-Ljungdahl pathway or the reductive acetyl-CoA pathway to reduce C1 compounds (e.g. CO, CO_2_ + H_2_, formate, methanol), into acetate, ethanol, butyrate, butanol, 2,3-butanediol, caproate, hexanol, and lactate as the products of this pathway [32]. In this pathway, different acetogens have been reported, such as mesophilic bacteria (e.g. *Clostridium* sp.), thermophilic bacteria (e.g. *Moorella thermoacetica*), mesophilic archaea (e.g. *Methanosarcina acetivorans*), and thermophilic archaea (e.g. *Archaeoglobus fulgidus*) [33]. The Wood-Ljungdahl pathway consists of two branches, the methyl and carbonyl branches, where several carbon reductions occur via distinct enzymes to form acetyl-CoA, a precursor of diverse products.

Starting from the methyl branch, CO_2_ is reduced to formate which, in a consuming ATP step, is catalyzed by forming 10-formyl-H_4_folate (HCO-THF). The conversion of HCO-THF to 5,10-methenyl-H_4_folate cyclohydrogenase (CH-THF) occurs previously then a NADPH-dependent reduction, where CH-THF is converted to 5,10-methylene-H_4_folate (CH_2_-THF). Subsequently, CH_2_-THF is reduced to (6S)−5-methyl-H_4_folate (CH_3_-THF). At the end of the methyl synthesis, the methyl group is transferred to a corrinoid iron-sulphur protein and reduced by a reduced ferredoxin forming methyl-CoFeS-P, finishing the reductions in the methyl branch [6, 25].

In the carbonyl branch, one molecule of CO enters the branch directly when CO is a sole substrate, and the other molecule is oxidized to CO_2_ by CODH, which is reduced in the methyl branch as explained previously. Otherwise, if CO_2_ is used as a sole substrate, CO_2_ is reduced to CO by the CODH/ACS complex, and the other molecule is reduced to methyl via the methyl branch [25]. The CODH/ACS complex is responsible for combining these two branches to form acetyl-CoA. The generated acetyl-CoA is the precursor of different end-products. To form acetate, a catabolic pathway occurs, where acetyl-CoA is converted to acetyl-phosphate and then transformed to acetate, where a previous study achieved 83% of the theoretical acetate production with mixed anaerobic culture in a syngas fermentation process [6, 25, 34]. To obtain ethanol, acetyl-CoA is reduced to acetaldehyde in the presence of aldehyde/alcohol dehydrogenase (Eq. 6) and then is converted into ethanol by the enzyme alcohol dehydrogenase (Eq. 7) [25]. The efficiency of ethanol production has been demonstrated by the continuous production by LanzaTech in their robust process [35]. However, it is not yet feasible to achieve high levels of ethanol titres in syngas fermentation as a lack of methodology hinders higher production and also some syngas fermentation process challenges, such as low productivity rates, and gas–liquid mass transfer [10]. For polyhydroxyalkanoate (PHA) production, two acetyl-CoAs are needed to form acetoacetyl-CoA, which is further converted to 3-hydroxybutryryl-CoA and polymerized to PHB (polyhydroxybutyrate), a class of short-chain length PHA [36]. The PHA content produced has been reported to represent 37 to 80% of the dry biomass in the monoculture process, although the microbial growth rate is slow and can limit the production [36]. In addition, during this pathway, the carbon that is fixed into biomass can be a source of protein for animal feed, which is of current interest within the industry with a patented process [37, 38].

6 $${\text{CH}}_{3}\text{COS}\bullet \text{CoA }+\text{NADH}+ {\text{H}}^{+}\to {\text{CH}}_{3}\text{CHO}+ {\text{NAD}}^{+}+\text{HS}\bullet \text{CoA}$$

7 $${\text{CH}}_{3}\text{CHO }+\text{NADH}+ {\text{H}}^{+}\to {\text{CH}}_{3}{\text{CH}}_{2}\text{OH}+ {\text{NAD}}^{+}$$

#### Methanogenic Pathway

The anaerobic formation of CH_4_ can be performed through H_2_, CO_2_, CO, formate, methanol, acetate, or methylamines. [23]. A previous study has observed a near total conversion of syngas to CH_4_, where a nearly pure CH_4_ stream was achieved representing 98% of the total headspace volume of the serum bottles used, with the main methanogenic microorganism represented by an archaea (i.e. *Methanothermobacter*) [19]. This high efficiency can be achieved as different pathways are also intermediates to produce CH_4_. In the biological WGS reaction (Eq. 2), H_2_ and CO_2_ which were converted from CO are further converted to acetate in the homoacetogenesis reaction (Eq. 8) and then to CH_4_ by the acetoclastic methanogenesis reaction (Eq. 9) or converted to CH_4_ in the hydrogenotrophic methanogenesis reaction (Eq. 10) [39]. From CO, CH_4_ can be directly converted by the carboxydotrophic methanogenesis reaction (Eq. 11) or first be converted to acetate by the carboxydotrophic acetogenesis reaction (Eq. 12) and then to CH_4_ by acetoclastic methanogenesis reaction (Eq. 9) [39].

8 $$4{\text{H}}_{2}+2{\text{CO}}_{2}\leftrightarrow {\text{CH}}_{3}\text{COOH}+2{\text{H}}_{2}\text{O}$$

9 $${\text{CH}}_{3}\text{COOH}\to {\text{CH}}_{4}+{\text{CO}}_{2}$$

10 $$4{\text{H}}_{2}+{\text{CO}}_{2}\to {\text{CH}}_{4}+2{\text{H}}_{2}\text{O}$$

11 $$4\text{CO}+2{\text{H}}_{2}\text{O}\to {\text{CH}}_{4}+3{\text{CO}}_{2}$$

12 $$4\text{CO}+2{\text{H}}_{2}\text{O}\to {\text{CH}}_{3}\text{COOH}+2{\text{CO}}_{2}$$

## Factors Affecting Syngas Fermentation

The use of pure strains as inoculum has been the main choice from diverse studies, as pure strains are more predictable and, consequently, more suitable for process optimisation compared with co-cultures or natural consortia. In the choice of natural consortium as inoculum, the inoculum conditioning process is necessary to avoid the activity of undesired metabolism. Furthermore, microbial metabolism can be affected by the interaction of different conditions in the process, including available nutrients, syngas composition, impurities, and process parameters such as temperature, pH, and pressure. Here, we focus on syngas fermentation for the biological conversion into H_2_, a potential biofuel and feedstock for diverse applications, and organic acids which are important chemicals mainly produced by non-renewable sources [26, 33]. Similar to the H_2_ generation from fossil fuels, which contains CO_2_ in its composition, H_2_ production from syngas can theoretically achieve a ratio of 50:50 (H_2_:CO_2_), which requires further separation, as will be discussed in the “Product Recovery and Purification” section. Organic acids’ production is influenced by the specific microorganisms present in the process and the environmental factors applied, which can result in different product concentrations. Here, we focus on the tools and strategies to increase the feasibility of the industrial production of H_2_ and organic acids, as these two products are still under-explored and have the potential to be produced on large scales.

### Inoculum Source

An alternative to monocultures is the use of synthetic co-cultures, through mixing two or more strains in one culture [40]. With designed co-cultures, there is a possibility to predict the activity of the microorganisms and modulate the process where strains can be replaced, added, or removed based on the process needs [41]. Moreover, when a co-culture is designed, the interaction mechanism in the microbial community, such as mutualism or commensalism, can favour the production preventing metabolite inhibition or even combining different microorganisms that can utilize different substrates [42]. However, if a co-culture is not well studied and/or designed, negative effects can be observed, as microbial interactions, such as parasitism, can impact the overall process [41].

Undefined, mixed consortia present more resilience to environmental factors and less aseptic requirements, being economically favourable for applications on an industrial scale [18]. However, their complexity and the possible undesired microbial interactions can reduce the yield of the desired products [43]. Nevertheless, to avoid the undesired microbial pathways activity, conditioning methods can be applied, as it is discussed next.

### Inoculum Conditioning

Conditioning strategies are extensively used in various bioprocesses, such as mechanical, thermal, chemical, and biological methods. Mechanical treatments can utilize approaches that physically alter the structure of sludge feedstocks. This occurs from mechanical force, such as ultrasonication or disperser methods, that produces sufficient shear forces that cause the disintegration of the sludge. During the ultrasonic treatment, the inoculum disintegrates, facilitating improved digestion, which can cause an increase in VFA production [44]. Moreover, applying two methods, ultrasonication and alkaline adjustment, with an increase of sludge hydrolysis and the inhibition of the methanogenesis via pH adjustment, increased VFA production [44]. Further strategies of combining methods resulted in a fourfold increase of H_2_ production using thermo-chemo-dispersion, at 80 °C, pH 10, and a mechanical disperser [45].

Thermal treatments can utilize cold treatment, for example, showing the effects of freezing/thawing on untreated waste-activated sludge. Liu et al. [46] froze sludge for 4 h at a temperature of − 5 °C and thawed it for 4 h at 20 °C together with nitrite addition. With this process, it was possible to increase the H_2_ production and suppress the activity of homoacetogens, methanogens, and sulphate-reducing microorganisms. Related to heat treatment, Liu et al. [43] have observed a fast CO conversion into H_2_ after heat treatment of 120 °C for 1 h in an anaerobic granular sludge, although the H_2_ produced started to be converted into CH_4_, showing that methanogens were not inhibited with this treatment.

Most chemical treatments are based on the alkalinity, where improvement could be achieved in two ways: (i) increasing the solubility of the organic materials in the sludge which can be easily utilized by acidogenic bacteria and (ii) decreasing the activity of the methanogens via unfavourable pH conditions [47]. Moreover, chemical methods can also use specific antibiotics/antimicrobial agents to inhibit the activity of targeted undesirable microorganisms (e.g. sodium 2-bromoethanesulfonic acid, BES; chloroform). Liu et al. [43] tested three different treatment methods: heat treatment, the addition of BES, and the addition of chloroform. The addition of chloroform was the treatment where H_2_ production was more efficiently converted with a stable production, showing a complete inhibition of methanogenesis and homoacetogenesis.

For biological treatments, enzymes, such as amylase and protease, or bio-surfactants, such as rhamnolipids, are commonly used to facilitate the hydrolyzation of organic matter [47]. Promoting the biodegradability of sludge with a biosurfactant (i.e. rhamnolipid) together with a thermal treatment, Chuanchuan et al. [48] could promote the enrichment of acid-producing microorganisms which increased VFA production by 33% compared with the thermal treatment.

In a long-term and/or continuous process, some conditioning methods might abate the inhibition capacity during the fermentation period, and a supplementary addition of chemicals could be necessary. However, selecting a pH and temperature range that can inhibit the undesired microorganisms can be an efficient and environmentally friendly tool to control the process without chemical addition [49].

### Inoculum Density

Besides the inoculum source, cell density can also impact syngas fermentation production. In a study with a pure culture (*Clostridium ljungdahlii*), Perret et al. [50] observed that by increasing the cell density by 2.5 times, the yield of the products increased by 1.5 times. Moreover, a production shift was observed, where with a higher cell density more ethanol was formed compared with a lower cell density where more acetic acid was produced. This can be explained by the response of bacterial quorum sensing, a bacterial communication mechanism, where an evaluation of the population density and the resources available is used to improve the adaptability of the organism in different environmental conditions [27]. Using natural consortiums, another study observed that an increase in inoculum concentration led to higher syngas conversion rates and, consequently, higher production rates [51].

### Medium Composition

Syngas provides the principal carbon and energy source, but supplementary minerals salts, trace metals, and vitamins are required to maintain cell growth and encourage product formation. Nitrogen is an essential nutrient for maintaining cell growth, and ammonia is the preferred source for H_2_ production [52]. Ammonia is an essential compound for ethanol production, as its absence can reduce microbial growth alongside ethanol production but has been reported to increase acetate production [53]. Different sources of ammonia can be used. A study has replaced NH_4_Cl with NH_4_OH and eliminated a buffer that accounted for 94% of the growth medium cost [54]. At pH below 10, NH_4_OH disassociate into NH_4_^+^ and OH^−^ acting as a base to raise the pH in syngas fermentation, eliminating the need for a buffer [54]. Phosphate is also an essential supplement, providing phosphorus for growth, and can be used as a buffering agent, maintaining pH [55]. The effects of other mineral salts are summarized in Table 1.

**Table 1:** Medium components and their impact on syngas production

Medium composition	Inoculum	Gas composition (CO:CO_2_:H_2_:CH_4_:N_2_)	Production impact	Reference
Mineral salts				
NH_4_^+^	*Clostridium ragsdalei*	70:6:0:0:24	In the absence, ethanol production and cell growth are reduced, and acetate production can be improved	[53]
Mg^2+^	*Clostridium ragsdalei*	70:6:0:0:24	Absence reduced ethanol production and increased acetate production	
Trace metals				
Zn	*Clostridium carboxidivorans* P7	50:35:15:0:0	Zn could increase the gene expression of carbon fixation and alcohol dehydrogenase and increase alcohol production	[56]
Fe	*Clostridium carboxidivorans* P7	50:35:15:0:0	A decrease in product synthesis and biomass accumulation was observed when Fe was absent	[57]
Ni	*Clostridium ragsdalei*	70:6:0:0:24	Necessary for microbial growth, acting on key enzymes, and can increase ethanol production	[58]
Co	*Clostridium ljungdahlii*	70:6:0:0:24	Ethanol production decreases when it is absent, but has no changes in higher concentrations	
Cu	*Clostridium ljungdahlii*	60:10:10:10:10	Increase ethanol and biomass production	[59]
Mo	*Clostridium carboxidivorans* P7	50:35:15:0:0	Important for CO utilization and can increase alcohol production	[57]
Se	*Clostridium ragsdalei*	70:6:0:0:24	Enhanced the activity of FDH with optimum concentration	[58]
W	*Clostridium ljungdahlii*	60:10:10:10:10	Increase ethanol and biomass production	[59]
Reducing agents				
Cys	Clostridium carboxidivorans	100:0:0:0:0	High biomass production and lower production of ethanol and butanol	[60]
Na_2_S	Clostridium carboxidivorans	100:0:0:0:0	High concentration of ethanol and butanol and low biomass production	

Trace metals are essential for microbial cell growth, as key enzymes that are responsible for CO oxidation, CO_2_ reduction, and H_2_ oxidation are enzymes containing metal ions [58]. As an example, zinc is related to regulating the expression of formate dehydrogenase, which can increase the amount of acetyl-CoA, due to an improvement in carbon fixation as the CO absorption and conversion can be increased by zinc [56]. Iron is possibly the most required metal for acetogenic cell function, as key enzymes and co-factors of the Wood-Ljungdahl pathway need iron for electron transfers [54]. Moreover, reducing agents also have an impact on the microorganism’s metabolism as observed by Lanzillo et al. [60], where sodium sulphide at an ideal concentration achieved the highest butanol and ethanol production, being the most effective reducing agent among the tested ones. Besides the improvement of microbial metabolism, various studies also have focused on the formulation of a cost-effective medium by decreasing and/or removing the components in the medium [61] and replacing them with cheap alternatives [62, 63].

### Syngas Composition

Depending on the biomass properties and gasification conditions (e.g. gasification temperature, pressure, and reactor design), the final syngas composition varies [14]. Syngas composition is also influenced by the process steps where the syngas is captured from industries’ emissions. In the conventional steel mill industries, three main off-gases are generated from different process steps: coke oven gas, which may contain large H_2_ content; blast furnace gas, which is the largest emission of CO_2_ in the plant; and blast oxygen furnace gas, which contains a gas rich in CO [64]. Studies have shown the impact of the gas composition on syngas fermentation (Table 2). Testing pure streams of CO or H_2_ and synthetic syngas, El-Gammal et al. [65] observed that independent of the type of inoculum, pure or mixture culture, CO as pure gas was the one that could achieve the highest VFA production. Conversely, with lower concentrations or even the absence of CO, an enhancement of acetate production was observed using *Moorella thermoacetica* [66]. A positive characteristic of mixed cultures is their great adaptability, where even with fluctuations in syngas composition, studies demonstrated their potential to produce acetate or ethanol [67]. Moreover, syngas composition can also impact the microbial community. Li et al. [19] performed experiments with six different syngas compositions using anaerobic sewage sludge and observed that the gas composition had little impact on the archaeal community, where *Methanothermobacter* was the main methanogenic organism. However, the bacterial community composition was noted to change with the gas composition ratios, with examples including the *Coprothermobacter* sub-population being significantly reduced when H_2_ was added in comparison to CO as the sole carbon substrate.

**Table 2:** Syngas compositions and effects on end-products

Inoculum	Gas composition (CO: CO_2_: H_2_: N_2_)	Main products (arranged in descending order)	Reference
CO-enriched mixed culture	100:0:0:0	Acetate, ethanol, biomass	[67]
	21:0:63:16	Acetate, biomass	
	21:21:0:58	Acetate, hydrogen, ethanol, biomass	
	21:9:12:58	Acetate, ethanol, biomass	
*Acetobacterium*-like isolate	100:0:0:0	Ethanol, hydrogen, acetate, biomass	
	21:0:63:16	Ethanol, hydrogen, acetate, biomass. *CO was not totally consumed	
	21:21:0:58	Acetate, ethanol, hydrogen, biomass. *CO was not totally consumed	
	21:9:12:58	Hydrogen, ethanol, acetate, biomass	
*Pleomorphomonas*-like isolate	100:0:0:0	Hydrogen, biomass	
	21:0:63:16	Hydrogen, biomass. *CO was not totally consumed	
	21:21:0:58	* CO was not consumed	
	21:9:12:58	Hydrogen, biomass. *CO was not totally consumed	
*Clostridium ragsdalei* P11	60:5:35:0	Acetate, other VFAs, ethanol	[65]
	100:0:0:0	Acetate, other VFAs, ethanol	
	0:0:100:0	Acetate	
Heat-treated digested manure	60:5:35:0	Acetate, other VFAs	
	100:0:0:0	Acetate, other VFAs	
	0:0:100:0	Acetate, other VFAs	
*Moorella thermocetica*	99.5:0:0:0.5	Acetate, biomass. *Prolonged lag phase without the total consumption of CO	[66]
	50:0:50:0	Acetate	
	41:18:41:0	Acetate	
	30:0:70:0	Acetate. *Shortest lag phase	

### Impurities in Syngas

Using raw syngas directly from heavy industrial processes, solid matter (e.g. ash), condensable volatile, and undesired gases are present [31]. Gas streams can contain impurities or contaminants, such as nitrogen-based compounds, sulphur-based compounds, xylenes (BTX), hydrogen fluoride, ammonia, and tars [7]. Some compounds can be beneficial towards microbial growth and production but largely are inhibitory and cause toxic effects [68]. Studies have been investigating the effect of these compounds on the metabolism of microorganisms in syngas fermentation (Table 3). A study has conducted a stepwise adaptation in syngas fermentation where the negative effects of BTX were overcome in different ranges of concentration, after the adaptation process with *Clostridium autoethanogenum* [16]. In another study, the impact of ammonium and nitrate concentrations, comparable to those observed in real syngas, was investigated. A decrease in the overall cell growth and alcohol production of some members of the *Clostridium* genus was observed, although some strains demonstrate greater tolerance than others at higher concentrations [69]. Ruckel et al. [68] showed a positive effect of ammonia and H_2_S addition with *Clostridium carboxidivorans*, improving growth, alcohol production, and CO consumption.

**Table 3:** Gas impurities and impacts on syngas fermentation

Inoculum	Gas impurities	Impurity impact	Possible gas treatment	Reference
*Clostridium carboxidivorans* P7^T^	Ash, tar, NO (150 ppm)	Cell dormancy and redistribution of production from acetic acid to ethanol in the presence of tar. Inhibition of hydrogenase enzyme by NO	Filter in the gas cleanup. Keep NO concentration below 40 ppm	[70, 71]
*Clostridium autoethanogenum*	Defined additions of NH_4_^+^, H_2_S, and NO_3_	Low tolerance to NH_4_^+^, reducing productivity. Strong inhibition of H_2_S. NO_3_ slowed the biomass formation and suppressed alcohol formation	Syngas purification to eliminate NO_3_ and keep the concentration of NH_4_^+^ and H_2_S in acceptable ranges	[69]
*Clostridium ljungdahlii*		NH_4_^+^ reduced productivity. Strong inhibition of H_2_S. High tolerance to NO_3_, alcohol formation was suppressed		
*Clostridium ragsdalei*		NH_4_^+^ reduced productivity. H_2_S in low concentration promoted biomass, acid, and alcohol production. NO_3_ slowed and suppressed the biomass formation and ceased alcohol formation		
*Clostridium carboxidivorans*	Defined additions of NH_3_, H_2_S, NO_3_-, and NO_2_-	NH_3_ and H_2_S improved CO consumption, cell growth, and alcohol production. NO_X_ suppressed growth and alcohol production	Reduce NO_x_ during the gasification process and/or remove it in another process	[68]
*Clostridium ragsdalei*	NH_3_ (mole fraction of 0.37%)	NH_3_ is converted to NH_4_^+^ by exposure to fermentation media. An accumulation of NH_4_^+^ leads to an inhibition of hydrogenase activity and cell growth	Remove NH_3_ from raw syngas	[31]

### Operational Parameters

Bioprocess parameters (e.g. pH, temperature, etc.) are important aspects that can improve the syngas fermentation process. pH can affect both the growth rate and the fermentation product [20]. Temperature also plays an important role in syngas fermentation, as gas components can be less soluble at high temperatures [17]. Moreover, increasing the pressure mass transfer and the solubility of the gas substrates are improved, benefiting cell growth and product formation [72]. In addition, with an appropriate gas flow rate, gas components can be more available for the microorganism’s uptake improving their production [73]. Moreover, parameters are intrinsically related to each other, as summarized in Table 4, then the optimum selection of these parameters should be based on the desired product and the cell metabolism.

**Table 4:** Summary of the operational parameters used in syngas fermentation and their products

**Inoculum**	**pH**	**Temperature (**°**C)**	**Pressure (bar)**	**Gas flow (vvm)**	**Liquid flow (mL/min)**	**Reactor**	**Products**	**References**
Undefined mixed cultures	6.0	37	–	0.005	–	TBR	Butyric and caproic acid	[74]
*Clostridium carboxidivorans* P7	5.75	33	–	0.008	–	CSTR	Ethanol, butanol, and hexanol	[75]
Enriched anaerobic sludge	8.5	28	2	–	–	High-pressure reactor	VFAs	[76]
*Thermococcus onnurineus*	6.5	80	7	0.4	–	BCR	Hydrogen	[29]
Anaerobic sludge	4.5	20	–	0.01	–	Experimental bioreactor	Acetate	[34]
Waste-activated sludge	7.5	35	–	–	–	CSTR	VFAs	[77]
Sewage sludge	9.0	20/37	–	–	–	–	Acetate	[49]
Domestic wastewater sludge	7.18	37	–	–	–	–	Acetate	[78]
*Clostridium ragsdalei*	–	32	–	–	–	–	Ethanol	[79]
*Clostridium autoethanogenum*	4.75	30	1.6	–	–	–	Ethanol	[72]
*Clostridium carboxidivorans*	4.72	–	2	0.01	–	High-pressure reactor	Ethanol	[80]
*Clostridium carboxidivorans*	–	35	1.1	–	–	–	Acids and alchols	[81]
*Thermococcus onnurineus* NA1	6.5	80	9	0.4	–	CSTR	Hydrogen	[73]
*Clostridium carboxidivorans* P7	6.0	–	–	0.4	500	MBR	Ethanol and acetic acid	[82]
*Clostridium carboxidivorans* P7	6.0	–	–	0.025	200	HFMBR	Ethanol and acetic acid	[83]

### pH

During fermentation, acidogenic bacteria can produce acids that accumulate and cause a pH drop, resulting in reduced microbial growth and a switch to a solventogenic phase, decreasing acetate concentration and increasing ethanol production [84]. The product can also be changed with a rise or decrease in pH values. A pH value of 6 was observed to favour the production of butyric and caproic acid in some strains [74]. To produce ethanol, butanol, and hexanol, a study demonstrated that a pH of 5.75 was more favourable than a pH of 4.75 to convert syngas into organic acids [75]. Another study had an enhanced VFA production at an alkaline pH (8.5) showing how pH can influence the microorganism’s metabolism [76].

To avoid a typical pH drop, Park et al. [29] controlled the pH value to 6.5 and subsequently achieved an H_2_ production 24% higher than without a pH control. A neutral pH is generally suitable for acetate production [33]. However, the pH values reported in the literature are not comparable as other process conditions can also impact syngas fermentation production. Acidic pH (4.5) has been reported to be favourable towards acetate production at 20 °C with anaerobic sludge, in which high acetate production was to be favoured by the inhibition of methanogens at this pH [34]. At alkaline pH, Liu et al. [49] reported the maximum VFA production with mixed culture from sewage sludge. Using a mixed culture from wastewater treatment, Nam et al. [78] observed the highest acetate production at neutral pH (7). In several of the presented studies, the values represent just the initial pH in a batch mode, without any control.

### Temperature

The definition of the temperature is based on the species of microorganisms that are being used for the process. However, in some cases, such as when mixed cultures with strains from different groups are used or when natural consortiums (with not identified species) are chosen, it is possible to select the temperature based on the conditions that were performed on the source (e.g. anaerobic digestor) or the temperature that favour the production of the selected product. Furthermore, in a process with mixed cultures or natural consortiums, the physicochemical conditions of the fermentation can potentially select microorganisms that can be more efficient and adapt to the environment [18].

As observed by Chandolias et al. [21] with a mixed culture fermentation with syngas, temperature exerts a significant influence on H_2_ production, where at 30 °C mainly a mix of VFAs was produced, and shifting the temperature to 60 °C, H_2_ and acetic acid were the main products. For acetate production, low and mild temperatures are also related to increased productivity [34, 49, 77]. Testing three different temperatures with a waste-activated sludge assisted with riboflavin, Liu et al. [77] observed that riboflavin could stimulate microbial diversity at mild temperatures (25 °C and 35 °C), but at 55 °C, this stimulation ceased, and the microbial community was changed by temperature, leading to low acetate production. Another interesting result was shown by Liu et al. [49], wherein a syngas fermentation with alkaline conditions at both temperatures of 20 °C and 37 °C similar VFA production was obtained. Moreover, it was observed that under lower temperatures, the activity of methanogens was lower, as well as the energy required for the heating of the process, having an economically positive impact. A study has observed that at 32 °C, the production of ethanol with *Clostridium ragsdalei* was stimulated, while, at temperatures above 37 °C, a decrease in cell growth and ethanol production was noted [79].

### Pressure

One of the possible ways to increase the solubility of the gas in the medium culture can be by increasing the partial pressure as shown in Eq. 13, which can describe the dependence of the gas transfer on the partial pressure of that gas in the substrate [85].

13 $$\frac{1}{{V}_{L}} \frac{{dN}_{G}}{dt}=\frac{{K}_{L}a}{H} ({p}_{G}- {p}_{L})$$

where *V*_*L*_ is the liquid volume in the reactor, *N*_*G*_ is the mole amount substrate from the gas phase, *K*_*L*_*a* is the volumetric mass transfer rate coefficient, *H* is Henry’s law solubility constant, and *p*_*G*_ and *p*_*L*_ represent the partial pressure in the bulk gas phase and the liquid phase. Thus, several studies have focused on increasing the partial pressure of syngas to increase the mass transfer ratio.

In batch experiments with *C. autoethanogenum* and CO as sole substrates, Abubackar et al. [72] observed that by increasing the initial pressure to 1.6 bar, ethanol production was enhanced. Using another strain (*C. carboxidivorans* P7^T^), Hurst and Lewis [80] observed that with an increase in the CO partial pressure from 0.35 to 2 bar, cell growth increased by 440%, and ethanol production changed from non-growth-associated to growth-associated. However, acetate production decreased with CO partial pressure above 1.06 bar, presenting a higher production at 0.70 bar. Lanzillo et al. [81] observed that the best ethanol and butanol production of *C. carboxidivorans* with CO as the sole substrate was at 1.7 bar. Besides the use of CO as the sole substrate, Katakojwala et al. [76] used CO_2_ and H_2_ with an enriched anaerobic sludge and observed an increase of 26% in acetic and butyric acid production with a 2 bar headspace pressure compared with the production at atmospheric pressure.

CH_4_ production can also be affected by CO partial pressure. Moreover, inoculum sources can present different tolerances to pressure. Using an anaerobic granular sludge, Jing et al. [86] observed that the inoculum tolerated a CO partial pressure of 0.5 bar without affecting CH_4_ production, whereas, in a study using digested sewage sludge to produce CH_4_, a CO initial pressure of 0.25 bar was related to causing serious inhibition to CH_4_ production [87].

To improve H_2_ production by *Thermococcus onnurineus* with CO as the sole substrate, Park et al. [29] observed that pH control is an important parameter when pressure is applied, as pH can be unstable under pressure. Stabilizing pH, the authors could increase the H_2_ production by 22% compared with the fermentation without pH control at 7 bar in a bubble column reactor. A previous study working with the same strain utilized a step-by-step increase in CO pressure to enhance the H_2_ production in a continuous agitated process [73]. Overall, the use of pressure can increase syngas fermentation production, although pressurized systems that can handle high pressures are expensive, requiring specific components, impacting directly the cost of production.

### Gas–Liquid Flow Rate

The assessment of gas–liquid flow rates has been employed to enhance mass transfer and the productivity of syngas fermentation. Comparing a *Clostridium butyricum* syngas fermentation with a co-fermentation of *C. butyricum* with *Saccharomyces cerevisiae*, Monir et al. [88] observed that an increase in the syngas flow rate led to higher ethanol production in both types of fermentation. However, there is a limit between the increase in gas–liquid flow rates and the increase in substrate conversion and production [82, 83]. Moreover, when gas flow rates are increased, a phase of the inoculum adaptation can be observed. First, there is a decrease in conversion efficiency by the microorganism, and then, the conversion rate is stabilized, returning to the previous conversion rate before the increase in the gas flow rate [73]. An evaluation of gas and liquid flow rates is important to understand the cell’s limitation to consume and convert syngas into products and to evaluate the better use of syngas and medium culture, improving the overall process and production costs.

## Design of Syngas Fermentation Reactors

**Fig. 2:**
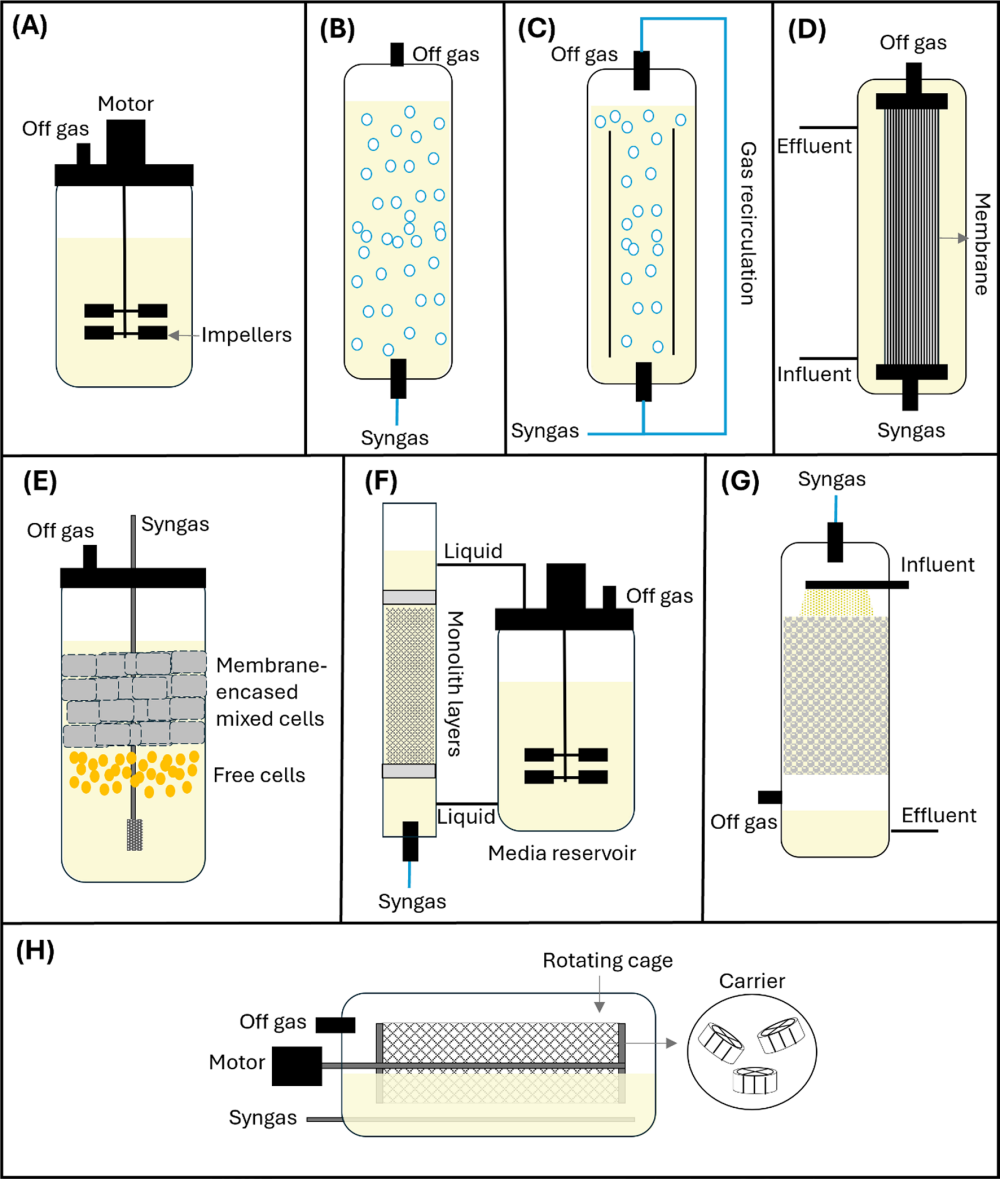
Reactors design applied for syngas fermentation. **A** Stirred tank reactor (STR). **B** Bubble column reactor (BCR). **C** Gas lift reactor. **D** Hollow-fibre membrane biofilm reactor (HFMBR). **E** Reverse membrane bioreactor (RMBR). **F**: Monolithic biofilm reactor (MBR). **G** Trickle-bed reactor (TBR). **H** Rotating packed bed biofilm reactor (RPBR)

The low productivity during syngas fermentation could be related to two factors: CO toxicity to microorganisms and CO low solubility, impacting the mass transfer rate [22]. The volumetric mass transfer rate coefficient (*k*_*L*_*a*) is an excellent parameter for comparing the mass transfer rates between different reactor designs. Since the main gas constituents have a low solubility (CO, H_2_, and CO_2_ are 28 mg/L, 1.6 mg/L, and 1.7 g/L, respectively), the mass transfer is one of the major bottlenecks of syngas fermentation production, and several studies have focused on the increase of *k*_*L*_*a* using different reactor designs [82, 89, 90]. The agitation rate is a valuable tool to increase the gas–liquid mass transfer as with higher agitation speeds, the gas–liquid interface is enhanced, although, for a commercial scale, agitated reactors are not economically viable as the energy input is high for mechanical agitation [7]. As illustrated in Fig. 2, there are several reactor design alternatives to increase the mass transfer rate during syngas fermentation. An alternative to increase gas solubility is an increase in partial pressure, as electrons from the gas substrate source can be more available for reduction reactions into value products [67]. Moreover, spargers also have a fundamental role in gas–liquid mass transfer efficiency, where with a reduced bubble diameter and an elevated gas flow rate, the gas–liquid interfacial area can be increased using microbubble spargers [89]. If an elevated gas flow rate is used, more gas is consumed, and the conversion rate will be low as the substrate supply can exceed the cells’ consumption capacity and product formation [82]. Then, a gas recirculation method should be used to avoid excessive gas consumption and improve the gas residence time [33]. Besides the gas–liquid mass transfer, cell density is also an important factor to improve syngas fermentation productivity. Strategies to increase cell density can also improve syngas fermentation, such as cell recycling, which can be done using hollow-fibre membranes, where high cell concentration can be achieved by retaining the microorganisms inside the reactor [91]. Moreover, cell retention can also be achieved by membranes using a hollow-fibre membrane biofilm reactor (HFMBR), where microorganisms can grow as a biofilm on the wall of the membranes and in addition have a higher mass transfer rate [89]. However, fouling is a recurrent problem that can occur in filtration units and membranes. Moreover, biofilm can also be formed in other types of reactors, such as monolithic biofilm reactors (MBR), trickle-bed reactors (TBR), and rotating packed bed biofilm reactors (RPBR), where a longer retention time for gas and nutrient uptake by the microorganism is provided by the direct interaction between syngas and the inoculum [33].

## Product Recovery and Purification

Downstream processes in syngas fermentation also face some hindrances, as the low concentration of the production, and the purification and storage of some products are still challenging. For example, to have the H_2_ biologically converted from the syngas fermentation process as a pure feedstock, the separation from CO_2_ is needed. From the theoretical production, H_2_ generated during biological WGS does not exceed 50% of the produced gas, with the remainder consisting mainly of CO_2_ [30]. Processes such as chemical absorption, cryogenic distillation, adsorption, and membrane separation can be used to purify the H_2_ produced. However, most of the methods are not suitable for biological processes as the requirements for feeding gas are higher (75–90%) than what is produced (~ 50%) [92]. A promising solution is the use of metal oxides for CO_2_ adsorption, where a study describes a high purity of H_2_ (> 90 mol%) via CO_2_ absorption in a high CO_2_ concentration (~ 50 mol%) [93].

VFA separation from the syngas fermentation broth is a further challenge due to the low concentrations and the requirement to separate a mixture of produced acids [94]. Moreover, it is beneficial to separate/remove VFA during production as accumulation can lead to a pH drop, resulting in the inhibition of microbial fermentation activity [95]. Different methods have been studied for VFA separation, such as membrane separation, electrodialysis, liquid–liquid extraction, adsorption/ion exchange, esterification, and nanofiltration [94, 95]. In a scalable way, membrane-based processes are the methods that can be used to increase the VFA recovery from bio-based processes [96]. With the aim to improve the environmental aspects of the electrodialysis (ED) technique, a study has used a bipolar membrane electrodialysis (BPMED) to extract acetate [97]. Even with a lower acetate flux than the ED process (< 15%), the BPMED process was more cost-effective (> 17%), and more environmentally friendly compared with the ED process. In a large-scale process design study, the authors have proposed three methods: vacuum distillation, pass-through distillation with vapour recompression, and pass-through distillation with multi-effect distillation, where high-purity isopropanol and acetone can be reached with a > 99% high recovery rate [98]. However, as highlighted by Sun et al. [95], the selection of the technique to recover VFAs should be based on the nature of the fermentation broth, the concentration of VFAs, and the posterior destination and purpose of the product.

Distillation is a long-standing traditional and widely used method for a variety of low molecular weight and volatile compounds. However, in a syngas fermentation process, the high energy cost related to the ethanol distillation process is still a challenge [10]. Different methods, such as pervaporation, membrane distillation, and vapour stripping membranes, have been employed for the separation of ethanol during syngas fermentation [99]. In a process designed for the recovery of bioethanol in a large-scale syngas fermentation process, a study has proposed vacuum distillation as the first step for ethanol recovery with further advanced separation and purification techniques that can recover 99.5% of the ethanol produced with high purity in a sustainable and economically viable process [100].

## Syngas Fermentation Process on an Industrial Scale

The diversity and abundance of gas feedstocks produced from non-food biomass and the relatively low demand for these gases make syngas fermentation an attractive tool for converting waste gases into valuable products. This has led to scaling up this process in some industrial plants around the world. During the early 2000s, commercialization plants started to be implemented by LanzaTech, Coskata, and Ineos Bio [35]. LanzaTech is one of the major players in the utilisation of syngas fermentation to produce ethanol. With a patented process and novel bacteria, ethanol production is already on a large scale, producing biofuel with high yields [35]. Their first commercial production plant inaugurated in 2018 in China has a reported annual capacity of 46,000 t of ethanol per year converted from steel mill emissions [101]. With a partnership with ArcelorMittal, the first facility to convert gases from steelmaking in Europe was inaugurated in 2022 in Ghent, Belgium, with a capacity of 125,000 t of generated ethanol annually [102]. Moreover, other companies and startups, with different levels of maturity (technology readiness level (TRL), [103]) have been using syngas fermentation to produce diversified products such as protein for animal feed, biofuels, biochemicals, and biomaterials (Table 5).

**Table 5:** Companies converting waste gases into products

Company	Plant location	Gas feedstock	Microbe	Product	TRL level in 2024
LanzaTech	China and Belgium	CO, CO_2_	*Clostridium autoethanogenum*	Ethanol	9
Visolis	USA	Biomass	Engineered microbes	Bio-based ingredients and materials	7
Aerbio	UK and Netherlands	CO_2_, H_2_	Non-GMO microbes optimized for protein production	Single-cell protein for the animal feed industry	7
Phase Biolabs	UK	CO2	Patented microorganisms	Ethanol	4
Mango Materials	USA	CH_4_	Natural selected microorganism	Biopolymers	5
Newlight Technologies	USA	CO_2_ or CH_4_	Natural selected microorganism	Biopolymers	9
Circe	USA	CO_2_	Engineered microbes	Fatty acids (for biopolymers and food)	7
Industrial Microbes	USA	CH_4_	Patented microorganisms	Biochemicals and biomaterials	7
Arkeon	Austria	CO_2_	Natural selected microorganism	Protein ingredients	8
Solar Foods	Finland	CO_2_	Natural selected microorganism	Protein ingredients	8
Again	Denmark	CO_2_	GMO bacteria	Acetate and acetic acid	6
Gas 2 Feed	Norway	CO_2,_ H_2_ and O2 from water electrolysis	Specialized microorganism	Protein ingredients	6

There is reason to speculate based on current trends that syngas fermentation will continue from laboratory research and be elevated towards a commercialized industrial process with a growing repertoire of bio-commodities. However, life cycle assessment (LCA) analysis, technical economical analysis (TEA), and modelling processes are necessary to estimate possible capital expenditures (CAPEX) and operational costs (OPEX), risks, and technical issues that can be evaluated before the commercial implementation. Most of the techno-economic assessments for syngas fermentation have been focused on ethanol production [104, 105]. Less attention has been on chemicals [106, 107], biopolymers, and H_2_ production from syngas fermentation [108]. However, other products also present the potential to achieve large-scale syngas fermentation production by exploiting microorganisms’ potential. Diverse products, such as alcohols, organic acids, biopolymers, H_2_, and single-cell protein, can be achieved from syngas fermentation, [11, 36, 109].

## Future Directions and Perspectives

Significant advancements have been achieved during the last decades to prove that syngas fermentation is feasible at a commercial scale. For the widespread of this technology, further research will be needed to cover the absence of knowledge in important subjects that were discussed in this review, besides the development of new technologies to enhance productivity. Reactor types have made substantial advancements in engineering techniques to overcome the solubility of syngas components, such as the utilization of pressure in various reactor configurations and the direct contact of the gas with microorganisms in a few reactor configurations (e.g. trickle-bed reactor). These techniques facilitate the consumption of syngas components by microorganisms. Moreover, it is fundamental to have all the risks and hazards analyzed, as syngas present in your composition a flammable and toxic gas, H_2_ and CO. The syngas composition is not only fundamental to accessing better productivity but also for the elucidation of the potential flammability of the syngas [110].

There is great potential to use natural consortiums in industrial applications. However, the metabolism understanding of these microorganisms and especially the interactions and microbial pathways on natural consortia are still in the early stages, making the comprehension of the conversion of syngas into different chemicals limited, not favouring the full potential production of this process [23]. Although selecting and applying appropriate conditioning methods may be a potential solution to address the knowledge gap in microbial interactions mediated by natural microbial consortia, complete inhibition of certain microbial pathways cannot be achieved solely through a single treatment approach, as it may not fully convert syngas into the desired product. The application of waste resources for conversion into valuable products is an important tool to efficiently meet environmental requirements and regulations for the climate change agreement, and syngas fermentation presents a valuable technology to achieve this goal in the near future.

## Conclusion

The search for sustainable products has advanced in recent years. Syngas fermentation shows the potential to produce diverse renewable products. However, the low yield of this process is still a drawback. By employing the methodologies presented and discussed in this review, the low yield and even the enhancement of production in a syngas fermentation process can be mitigated. Furthermore, this review presents a simplified understanding of the microorganisms’ metabolism, which is subsequently complemented by the application of engineering tools to control the process environment and optimize production conditions. In general, a process design is of extreme importance, defining the final product first will facilitate the next procedures, such as the determination of the necessary conditioning methods, the appropriate physiochemical properties of the process, and the selection of a suitable reactor to enhance the production and utilization of resources during syngas fermentation. A more comprehensive understanding of the microbial interactions and potential pathways within natural microbial consortia is imperative. Furthermore, the exploration of novel conditioning treatment methods and optimizations is necessary. The application of natural microbial consortia holds great potential for industrial applications, particularly in the field of syngas fermentation, which could enhance its commercial viability.

## Data Availability

No datasets were generated or analysed during the current study
